# Nanopore Current Events Magnifier (nanoCEM): a novel tool for visualizing current events at modification sites of nanopore sequencing

**DOI:** 10.1093/nargab/lqae052

**Published:** 2024-05-20

**Authors:** Zhihao Guo, Ying Ni, Lu Tan, Yanwen Shao, Lianwei Ye, Sheng Chen, Runsheng Li

**Affiliations:** Department of Infectious Diseases and Public Health, Jockey Club College of Veterinary Medicine and Life Sciences, City University of Hong Kong, Hong Kong, China; Tung Biomedical Sciences Centre, City University of Hong Kong, Hong Kong, China; Department of Infectious Diseases and Public Health, Jockey Club College of Veterinary Medicine and Life Sciences, City University of Hong Kong, Hong Kong, China; Department of Infectious Diseases and Public Health, Jockey Club College of Veterinary Medicine and Life Sciences, City University of Hong Kong, Hong Kong, China; Department of Infectious Diseases and Public Health, Jockey Club College of Veterinary Medicine and Life Sciences, City University of Hong Kong, Hong Kong, China; State Key Lab of Chemical Biology and Drug Discovery and the Department of Food Science and Nutrition, The Hong Kong Polytechnic University, Kowloon, Hong Kong SAR; Department of Infectious Diseases and Public Health, Jockey Club College of Veterinary Medicine and Life Sciences, City University of Hong Kong, Hong Kong, China; Tung Biomedical Sciences Centre, City University of Hong Kong, Hong Kong, China; Department of Precision Diagnostic and Therapeutic Technology, City University of Hong Kong Shenzhen Futian Research Institute, Shenzhen, Guangdong, China

## Abstract

Summary: Nanopore sequencing technologies have enabled the direct detection of base modifications in DNA or RNA molecules. Despite these advancements, the tools for visualizing electrical current, essential for analyzing base modifications, are often lacking in clarity and compatibility with diverse nanopore pipelines. Here, we present Nanopore Current Events Magnifier (nanoCEM, https://github.com/lrslab/nanoCEM), a Python command-line tool designed to facilitate the identification of DNA/RNA modification sites through enhanced visualization and statistical analysis. Compatible with the four preprocessing methods including ‘f5c resquiggle’, ‘f5c eventalign’, ‘Tombo’ and ‘move table’, nanoCEM is applicable to RNA and DNA analysis across multiple flow cell types. By utilizing rescaling techniques and calculating various statistical features, nanoCEM provides more accurate and comparable visualization of current events, allowing researchers to effectively observe differences between samples and showcase the modified sites.

## Introduction

Oxford Nanopore Technologies (ONT) sequencing has found extensive use, supporting a diverse range of applications, including genome assembly, full-length transcript detection, and base modification detection (1). This sequencing technology is based on the measurement of current signals as the RNA or DNA molecule passes through the nanopore. Early studies identified that modifications on DNA or RNA molecules influenced the ion current (2,3), discerned by comparing the current signals of modified and unmodified reads derived from the same sequence. Based on these findings, several methods have been developed to detect DNA/RNA modifications (4,5).

These tools generally compute various statistical features for each nucleotide based on corresponding current signals, often referred to as ‘events’. These features may include measures such as median, mean, standard deviation, and dwell time - the duration of time that a nucleotide spends passing through the nanopore. A process known as ‘resquiggle’, which identifies current events, has been developed and is widely used. Two well-known programs in this field are nanopolish (6) and Tombo (7). Furthermore, f5c, an optimized version of nanopolish that leverages GPU acceleration for improved performance, has been introduced recently (8). Following the resquiggle process, signal features can be calculated and incorporated into downstream modification detection tools such as Tombo (7), Nanom6A (9), m6Anet (10), Nanocompore (11) and xPore (12). In addition to that, some other modification detection methods like DeepMod2 (13) used the events indices generated from nanopore basecaller (Guppy/Dorado), which we refer to as the ‘move table’.

Given the demonstrated association between modifications and current events, a visualization tool exaggerating current signal features is crucial for deducing modified positions. Existing tools such as SquiggleKit (14), squigualiser (15), UNCALLED (16) and BulkVis (17) are designed for visualizing raw electric currents. However, the raw signal could be messy, making it difficult to observe current differences, particularly in high-coverage regions (Supplementary Figure S1A). Some modification detection tools, like Tombo and Nanocompore, can be used to visualize processed features of current signals. However, their plotting functions are coupled with genome-wide data processing, and thus cannot be used for general-purpose current event visualization, especially when focusing on target regions. Moreover, the normalization or rescaling of the raw current signals in the two pipelines is tailored for downstream analysis, not for visualization, making some of their visualization hard to interpret. Currently, ONT is maintaining multiple types of raw signals from different chemical nanopores, including the DNA sequencing data from R10.4.1 and R9.4.1 flow cells, and the direct-RNA sequencing data using R9.4.1 flow cells. A general-purpose visualization toolkit for processed statistical features in ONT DNA and RNA sequencing is needed for users who want to know the effect of epigenetic variations on current signals.

One additional aspect used for Nanopore DNA/RNA modification detection is the mismatches between reads and references. Sometimes the basecalls from nanopore raw signal would create specific errors due to the modifications. For example, in direct-RNA sequencing, EpiNano has demonstrated that alignment features can accurately predict m6A modifications (18). And the DNA modification in bacterial DNA is creating G to A mismatches in Nanopore basecalls (19). Exploring the alignment features would facilitate the development of DNA/RNA modification finding programs and help to validate the potential modification sites.

To this end, we developed nanopore Current Events Magnifier (nanoCEM), a Python command line tool to visualize alignment features and multiple current signal features of nanopore sequencing data. We also conducted statistical analysis to measure the observed signal differences. The nanoCEM, which can be used for both RNA and DNA analysis, currently supports the f5c resquiggle, f5c eventalign, Tombo and ‘move table’. It also incorporated a rescaling process to facilitate the comparison of relative signal change. As a result, the nanoCEM is a good visualization tool for showcasing and validating the differences between samples.

## Materials and methods

### nanoCEM pipeline overview

**Figure 1.**
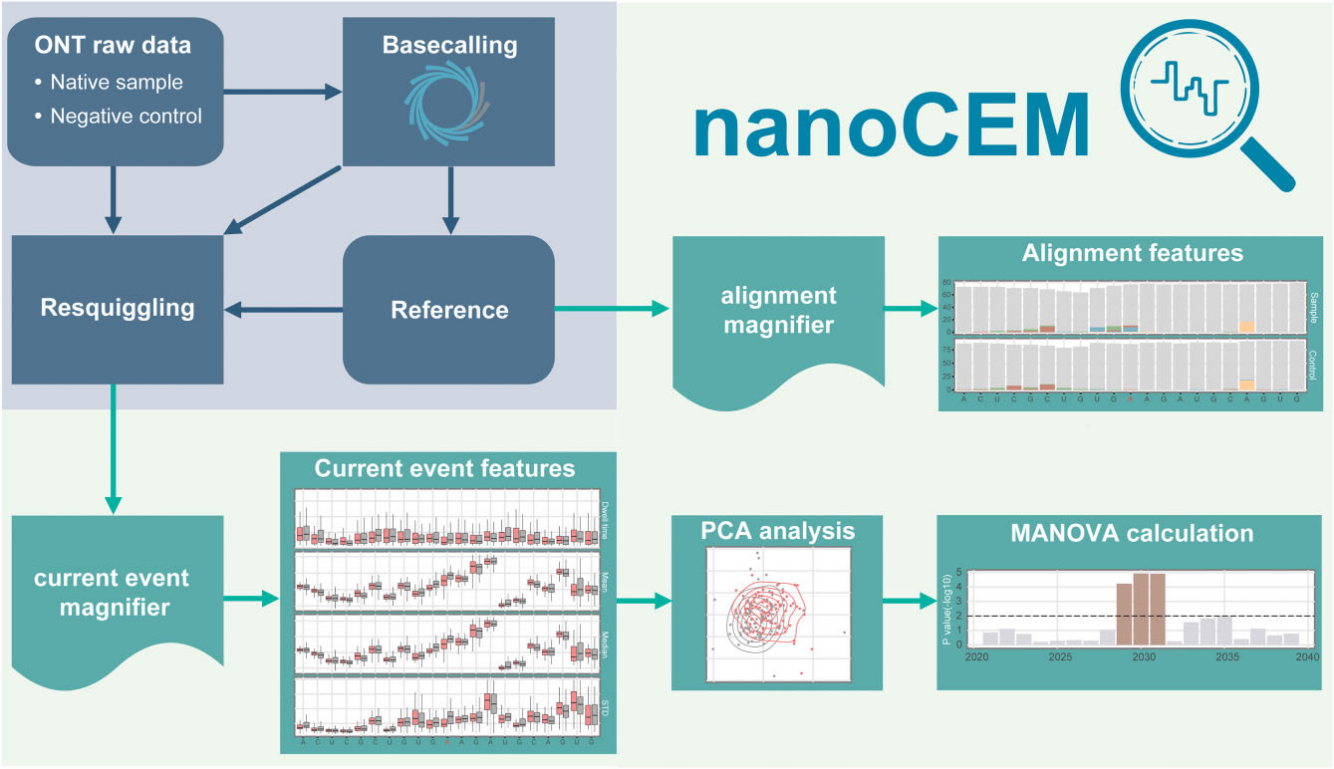
The workflow of nanoCEM. This diagram illustrates the process starting with basecalling of raw reads data using the ONT basecaller (blue background) and proceeding to the nanoCEM analysis (green background). The alignment magnifier takes the basecalled reads and reference sequence as input to produce an alignment feature figure. Concurrently, the current event magnifier, utilizing f5c resquiggle (default), creates a current feature figure. Subsequently, PCA analysis generates a scatter plot, visually contrasting two groups at the target site. Finally, MANOVA assesses the significance of the difference between two groups of each site in the target region.

The nanoCEM is a Python command line tool designed for validating high-confidence DNA and RNA modification sites based on the nanopore sequencing data. It accomplishes this by visualizing alignment features and current features. Additionally, it supports statistical analysis on the current feature to measure the observed differences. An overview of the main workflow is presented in Figure 1. Multiple files are required as inputs for nanoCEM, including a raw data file, a basecalled sequence file, and corresponding reference sequences. It is recommended to use transcripts sequences as the reference for RNA analysis due to the presence of RNA splicing, whereas genome sequences are suggested for DNA analysis. Typically, two datasets, a native sample and a low/no modification sample as the negative control, should be included in the nanoCEM pipeline for comparison.

The initial step of alignment feature visualization entails utilizing minimap2 (20) to align the basecalled reads to the reference sequence. Subsequently, ‘samtools mpileup’ (21) is employed to extract a special format alignment string of each target position, generating an intermediate file containing the information about nucleotide compositions at individual sites. Following the regular expression processing, the counts of ATCG at each position are logged and employed for visualization. To highlight mismatches, the matching bases are depicted in gray color in the generated bar chart.

During the analysis of current features, the f5c resquiggle (8) as the default preprocessing method is utilized to determine the current event index, followed by an embedded rescaling step. After obtaining the rescaled signals and event index, various features, including mean, median, standard deviation, and dwell time, are computed for each current event in the target region. Subsequently, these features are assigned to corresponding reference sequence. The resulting current event feature table is saved and utilized for generating box plots and violin plots. For statistical analysis, 3-mer features from two datasets are subjected to Principal Component Analysis (PCA) to discern potential distinctions of the target region, and only the centre position is visualized in a dot plot. Multivariate analysis of variance (MANAVO) is then applied to leverage the PCA outcomes and calculate P-values, indicating the statistical significance of observed variations. The generated P-values are stored and plotted as a bar chart, providing a visual representation of the significance level for each position within the target region.

### Key features

#### A pipeline for showcase and validation of candidate modification sites

Modification sites are usually inferred by comparing the alignment or current features of a native and a modification-free/deficient sample. nanoCEM is designed to visualize and validate these differences at target sites. For a given list of potential modification sites, nanoCEM can conveniently output a collective figure depicting the alignment and current features of two samples at the tested positions. The current differences can be further determined via PCA analysis, providing a statistical validation of the prediction results.

#### Extraction and normalization of current signals

The current signal information is stored in the nanopore raw sequencing data. With the introduction of R10.4.1 flow cells, the diversity of ONT data formats has increased. These now include the original single and multiple fast5 formats, the latest pod5 format, and the community-driven slow5/blow5 formats (22). NanoCEM supports blow5 by default. Therefore, format conversion is required if the starting data is not in blow5, which can be converted by slow5tools (23) or blue-crab (https://github.com/Psy-Fer/blue-crab). The relationship of different data formats and corresponding conversion tools is shown in the Supplementary Figure S1B.

In the preprocessing of ONT current signals, Tombo, nanopolish, and f5c have been widely used in different tasks. However, there are some differences between the three pipelines. Tombo, f5c eventalign and nanopolish eventalign aligns current signals to reference sequences, while f5c resquiggle aligns the signals back to the basecalled sequence. Furthermore, nanopore basecallers, such as Guppy and Dorado, have the capability to output move tables in basecalled BAM files. These move tables record the event indices aligned to the basecalled sequence like f5c resquiggle. To align event indices from f5c resquiggle and move table from basecaller to the reference sequence, we employ the BAM file generated from Minimap2 by mapping reads to the reference, as depicted in Supplementary Figure S1C.

Additionally, different preprocessing methods are found to have base shifting in signal alignment. And some offsets always exist between the results of f5c (including f5c resquiggle and f5c eventalign) and Tombo. Tombo has a built-in function to find the most contributing base, while f5c does not. The direct-RNA sequencing data (RNA002) is sequenced from the 3′ to 5′ direction, which made the offset between Tombo and f5c significant in some cases. Take a 5-mer CUAAG as example, f5c assigns the current event to the first base, which is C (**C**UAAG), while Tombo to the second one, which is U (C**U**AAG). To make f5c results comparable to Tombo and move table, We have provided the ‘–base_shift’ option in nanoCEM, with the default setting being ‘auto’ to perform this shifting operation for f5c using the shift table defined by squigualiser (15). After the automatic adjustment, the current signal alignments from different tools become more similar to each other.

Signal normalization can help reduce technical biases in sequencing and enhance the comparability of data. Specifically, the raw signal corresponding to a mapped section of a read is normalized using median shift and MAD (median absolute deviation) scale parameters. To reduce the impact of current noise, we implemented a thresholding approach whereby the threshold value replaced any normalized signal values exceeding a predetermined threshold (default: ±5). The equation for the normalization is:


\begin{equation*}NormSignal = \frac{{RawSignal - Shift}}{{Scale}}\end{equation*}

#### Statistical analysis based on current event features

After generating the current event feature table, the visual observation of discrepancies in the medians of various features at the same position, using boxplots and violin plots, imply differences. However, it is crucial to acknowledge that these visual disparities can be subject to errors. Therefore, additional statistical tests are necessary to provide more robust and reliable support for any observed distinctions.

We employed 3-mer current features to perform PCA analysis and identify whether it can address distinct distribution patterns between the two groups on the target site, of which we are primarily concerned. Then to provide a significance level of difference, MANOVA, which stands for Multivariate Analysis of Variance, is used to assess significant level when comparing each position between the native sample and negative control.

However, using 3-mer features may also result in considering two adjacent bases to be significantly different. Therefore, it is essential to manually identify the central points for continuous regions with high significant levels. Alternatively, if the motif is known, it can be employed to determine the modified site.

#### Support for RNA/DNA and multiple flow cells

f5c (v1.4) has been enhanced to support new R10 flow cell and RNA004 kit using ‘–pore’ option and ‘–rna’ for RNA support. Therefore, by incorporating support for f5c and blow5, we have achieved compatibility with different flow cells and DNA or RNA. Also, the ‘move table’ from ONT basecaller can also be used for new R10 flow cell and RNA004 kit which is also supported by nanoCEM.

The ONT RNA and DNA current signals are stored in different strand orientations. The RNA signal is currently stored from 3′ to 5′, in a reversed direction compared to DNA. We have made two optimizations for direct-RNA sequencing. First, the sequencing direction is from the 3′ end to the 5′ end. After the preprocessing step, it is essential to reverse both the signal and the related index before aligning them to the reference genome. Second, when generating plots using nanoCEM, it is customary to replace the nucleotide ‘T’ with ‘U’ to represent the RNA-specific base pairing accurately. Our default model is DNA, and we provide the option ‘–rna’ to switch to the RNA mode in nanoCEM.

#### Additional support and script for Tombo

Tombo, being a former modification detection tool, is still widely used. To support Tombo, we have made extensive adaptations. However, the installation process can be cumbersome due to the outdated environment and lack of updates for Tombo. Therefore, it is necessary for the user to independently install and configure the Tombo environment. Follow its pipeline, utilizing single fast5 data format data, and perform resquiggle. After Tombo resquiggle, the event index and corresponding reference position will be written back to the fast5 files. nanoCEM will extract this information from the fast5 files and perform the same operations as in the previous pipeline.

Tombo resquiggle is a time-consuming process that relies heavily on I/O. For the users who only want to analyze target regions using nanoCEM, a CLI script named ‘extract_sub_fast5_from_bam" is provided to extract the raw current files (fast5) mapped to a specific genomic region. This script supports single fast5 data format only.

## Results

### nanoCEM visualized the alignment and current features of RNA and DNA modifications

**Figure 2.**
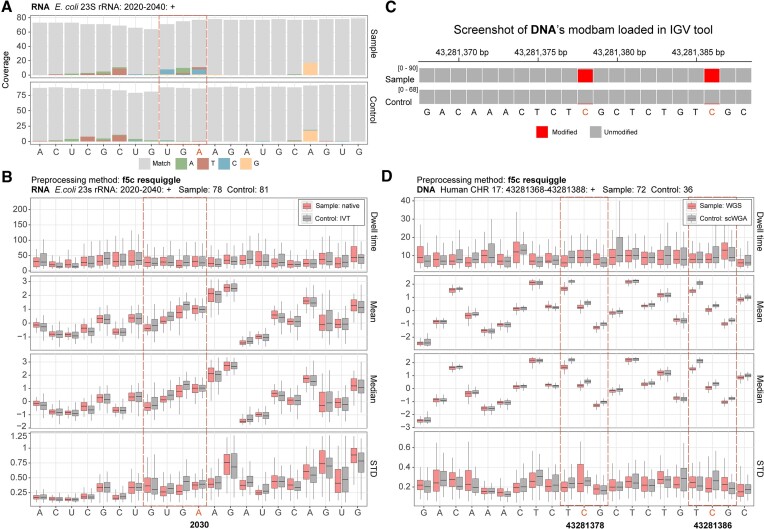
Feature visualization of nanoCEM. A and B present data from the ONT direct-RNA sequencing of the A2030 site in *E. coli* 23S rRNA, using R9.4.1 flowcells. (**A**) The alignment feature figure, contrasting native *E. coli* RNA (wild type sample) and IVT (*in vitro* transcribed RNA, serving as a negative control). Mismatches are highlighted, with matching bases shown in gray. (**B**) Current features, including dwell time, mean, median and STD, from the native and IVT RNAs are shown as red or black, respectively. (C, D) examine data from ONT DNA sequencing data of two CpG sites on chromosome 17, from human cell line HCC78. (**C**) The modified ratio on WGS (whole genome shotgun, native DNA) and scWGA (single cell whole genome amplification, amplified DNA with no modifications). (**D**) The current events feature visualization for the two CpG sites from WGS and WGA sequencing. The significant differences between negative and native samples are highlighted in the red box.

The A2030 site is a known methylated site (N6-methyladenosine, m6A) on the 23S rRNA of *Escherichia coli*. We used nanoCEM to visualize this modification by plotting the nanopore direct-RNA sequencing alignment and current features of a native sample and an *in vitro*-transcribed (IVT) equivalent, which serves as the negative control. Its neighboring nucleotides were also shown with ten flanking. The RNA modification can result in errors during basecalling, as the modifications will change current signals. The alignment errors from RNA read mapping, together with the change of the current features, can be used to differentiate the RNA modifications in direct-RNA sequencing (Figure 2A and B).

Unlike RNA, DNA modifications typically do not result in errors during the mapping process. Therefore, in the case of DNA modification, only current feature plots are recommended for output. For the DNA showcase, we ran nanoCEM for a pair of R9.4.1 DNA datasets. One data was from a whole genome shotgun (WGS) native sample, and another was from single-cell whole genome amplification (scWGA) sequencing. In theory, scWGA reads were generated from the amplified DNA without 5mC and can thus be used as a negative control for WGS reads. In a previous study (24), we demonstrated the presence of two 5-methylcytosine (5mC) sites on chromosome 17 using Megalodon by comparing the WGS and scWGA reads (Figure 2C). Via nanoCEM visualization (Figure 2D), the current changes caused by 5mC at these two sites were readily observed. Notably, the current features of the adjacent nucleotides (covering approximately a 3-mer region) were also impacted, especially the mean and median signal intensity values.

In our *E. coli* RNA dataset, no m6A RNA modification sites were located inside homopolymers. And we do not have false positive m6A calling related to homopolymer regions. Instead, we showcased two polyG regions, with or without RNA modification. One is the polyG region from 2250 to 2253 (Supplementary Figure S2, A to D), which includes G2251, a known 2′-O-methylation site in *E. coli* 23S rRNA. Notably, the standard deviations (STDs) in the polyG regions were higher than the neighboring regions, indicating a lower confidence and higher error rate in the comparison between *E. coli* IVT and native 23S rRNA samples using all four preprocessing methods. The other is an unmodified polyG region (2523 to 2526, Supplementary Figure S2, E to H). No noticeable differences were observed between *E. coli* IVT and native 23S rRNA samples. The RNA modification finding relying on current feature comparison in homopolymer regions could still be effective.

For DNA modification, we only screened the 5mC modification in the CpG site, which may not be a homopolymer-related motif. We selected a sequence containing two homopolymer regions to showcase our visualization function in nanoCEM. One is polyG (CHR17:43 281 920–43 281 923), and the other is polyC (CHR17:43 281 927–43 281 932) (Supplementary Figure S3). Unlike the polyN region in RNA, the polyN regions in DNA have a similar current level for each nucleotide, with a very low STD. However, the dwell time for each nucleotide could be quite different.

### Statistical analysis validated differences at target positions

**Figure 3.**
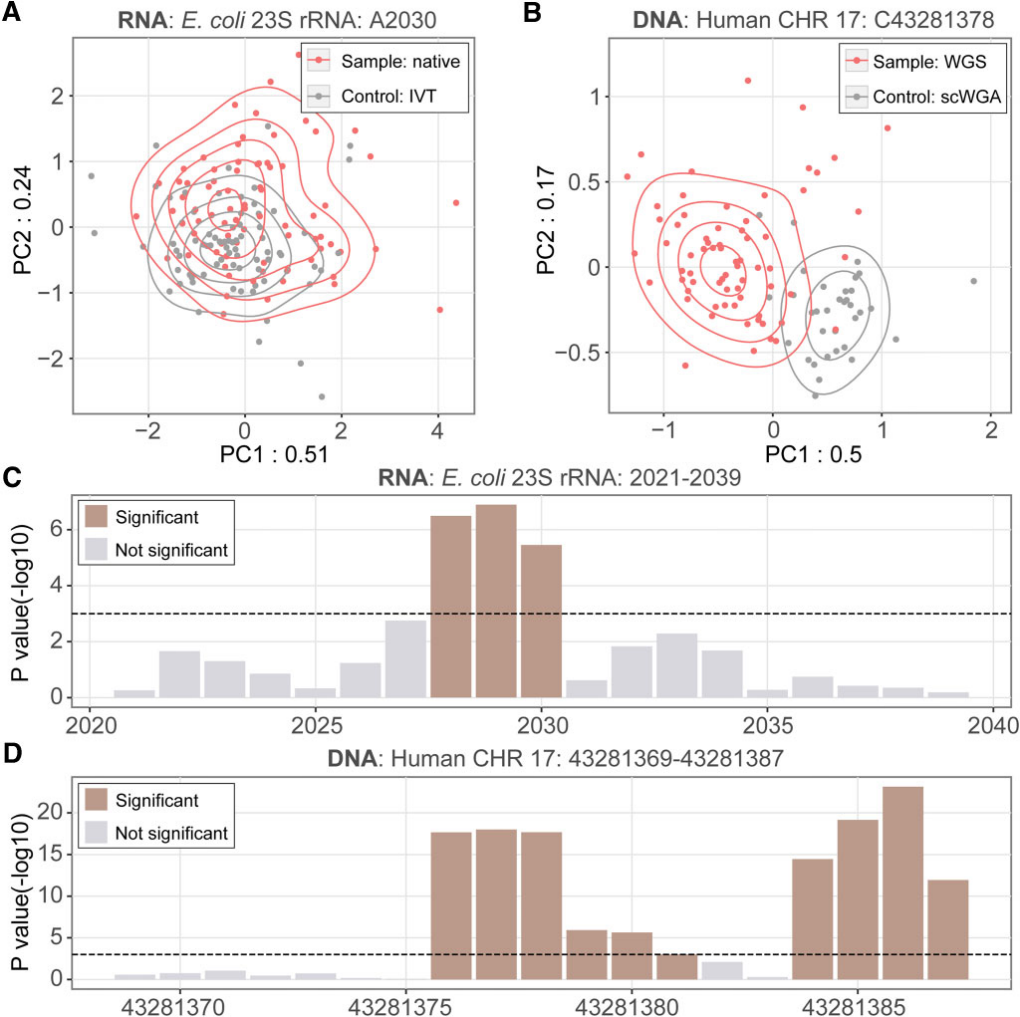
Statistical analysis with nanoCEM. The scatter plot of PCA represents site on RNA. (**A**) *E. coli* 23S RNA A2030 and DNA. (**B**) Human chromosome 17: 43 281 378 (a CpG site). (**C**, **D**) depict their significance levels determined by MANOVA in the target region, with -log10 p-value indicating the degree of significance. The dashed line indicates a *P* value = 1e-3.

Visualization facilitates direct observation of alignment and current differences between the native and negative-control samples. On this basis, nanoCEM also provides a statistical analysis function to measure the significance of these differences. Since nucleotide modifications may impact the current features of neighboring sites (Figure 2B and D), we utilized 3-mer in analysis, containing 3-mer × 4 features, to conduct PCA analysis. The results, as depicted in Figure 3A and B, revealed distinct distribution patterns of the modified and unmodified nucleotides. To provide further statistical support, we employed MANOVA to calculate significance values for the differences revealed by PCA analysis. Using a cutoff of −log_10_*P*-value at 3, it successfully identified the statistically different positions within the target region (Figure 3C and D).

We have provided an option ‘–kmer-size’ to allow the user to choose different *k*-mer lengths for downstream analysis. Here we used 3-mer in our PCA and MANOVO based on our observations of the difference between the negative control and native sample. For DNA, on 5mC sites, we can observe significant current differences between WGA and WGS reads for nucleotides C and G, together with the T before C, with all preprocessing methods (Figure 2D, Supplementary Figure S4C, Supplementary Figure S5C, and Supplementary Figure S6C), while the other nucleotides show no difference. For RNA, the three nucleotide differences can also be observed between IVT and native RNA samples around the 6mA site at the *E. coli* 23S 2030 position (Figure 2B). So, the default kmer-size is 3.

Collectively, the nanoCEM statistical analysis function successfully verified the locations of modification sites from both a visualization and a statistical perspective. However, the statistically significant positions are usually not restricted to the authentic modification site due to signal shifting and using 3-mer features. The authentically modified nucleotide is expected to be in the middle of the clustered region.

### Further exploration of nanoCEM result table

**Figure 4.**
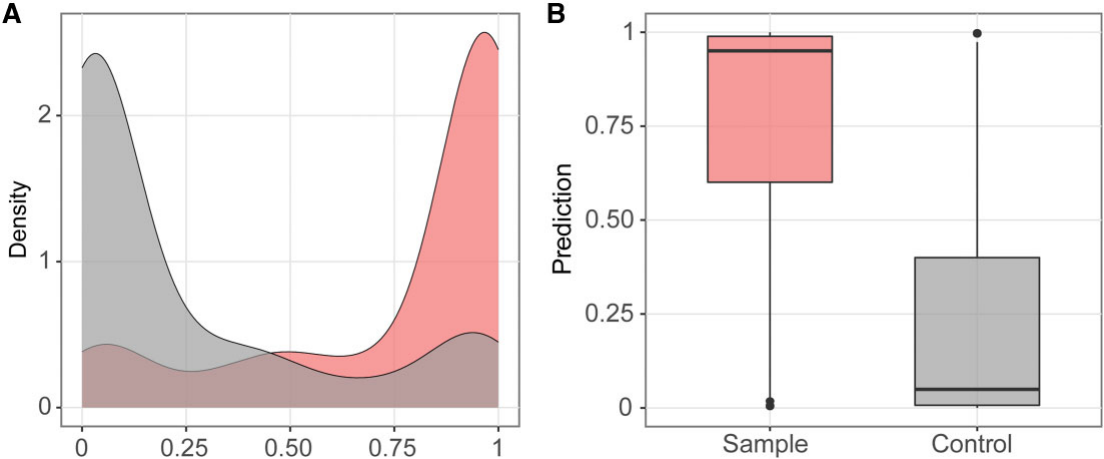
A demo showing applying machine learning approaches to predict RNA modifications from the output of nanoCEM. The XGboost predictor is trained using the A2030 current features. The prediction accuracy on the test dataset is visualized using (**A**) boxplot and (**B**) density plot.

After statistically confirming the current differences at the target site, we extracted the 3-mer current features (mean, median, STD, and dwell time) at this position and output a table containing 3-mer × 4 features. This table can be a resource for further machine learning training. Taking the *E. coli* 23S rRNA A2030 site as an example, we allocated 20% of the table as the testing set and 80% as the training set. We employed an XGBoost method to train a new predictor for the m6A with 5-fold cross-validation. The resulting accuracy was approximately 0.8, and the distribution of predictions on testing set can be observed in Figure 4.

These results demonstrate that the nanoCEM’s outputs can be effectively used as training input for machine learning algorithms, providing a framework for researchers who want to train their own DNA/RNA modification predictors. And the visualization functions, together with the well documented feature tables, can help the users understand the characteristics of modification sites, and help them to optimize the cutoff to be used in the modification predictors.

## Discussion

Comparing alignment features is a crucial technique for detecting RNA modifications in nanopore sequencing studies. The Nanopore basecall models have an impact on the read accuracy and alignment features, which should be considered for users who want to make DNA/RNA modification predictions using alignment features. For instance, the model used for ONT direct RNA sequencing could be ‘RNA_r9.4.1_70bps_hac’ from Guppy basecaller or ‘rna002_70bps_hac@v3’ from the latest Dorado basecaller. We compared the read mismatch ratios at the *E. coli* 23 rRNA A2030 site using Guppy (Figure 2A) or Dorado (Supplementary Figure S7). For both basecallers, we observed an increase in mismatch ratio near A2030, and a decrease in mismatch ratio of unmodified sites. This observation suggests that the new model still have similar alignment feature as the older one for the m6A detection using ONT direct-RNA sequencing. And nanoCEM can help the users to better access the performance of different models/basecaller regarding the read accuracy, facilitating the development of RNA modification detecting pipeline on ONT platforms.

While nanoCEM can offer valuable insights based on current event features, it is crucial to note that the presence of modified bases can influence neighboring bases and lead to fluctuations in the current levels. Therefore, it is often necessary to consider a wider window that includes additional 3–4 bases when performing nanoCEM analysis. The inclusion of multiple bases in the analysis window allows for a more comprehensive assessment of the context surrounding a potential methylation site.

For DNA and RNA modification showcase, three other preprocessing methods were used to verify nanoCEM (Supplementary Figures S4–S6). In the DNA showcase, although the move table generated by the basecaller differs in shape compared to the other three methods (Supplementary Figure S5C), the difference between the native sample and negative control at these two points is noticeable. Consequently, all four preprocessing methods produce very similar results and successfully identify two 5mC sites in the target region.

Nanopore reads are known to have a high error rate inside homopolymer regions. Our current feature visualization tool for these regions, can help validate the basecalls and facilitate downstream analysis. nanoCEM can show current differences between the native sample and the negative control inside homopolymer regions, which could be attributed to modifications. However, these differences could also be influenced by various factors, including errors introduced in preparing negative samples, like the DNA whole genome amplification (WGA) errors from PCR or *in vitro* transcription (IVT) errors from cDNA and RNA synthesis.

Apart from that, there can be other differences observations from different preprocessing methods. The known modification site is 2030 (Figure 3C). However, in our MANOVO test, the most significant site could sometimes shift to 2029 with different preprocessing method (Supplementary Figure S4B). We have also observed a dramatic change in current features around the modified CpG site in DNA sequencing data (Figure 3D). Furthermore, although differences can be observed in the current feature visualization using Tombo (Supplementary Figure S6A), the results of the MANOVA analysis are not statistically significant (Supplementary Figure S6B).

The base shift is an optimization we implemented for f5c, including f5c resquiggle and f5c eventalign. It ensures that the f5c result exhibits a similar trend compared to the move table and Tombo. In addition to R9 DNA and RNA (Figure 2), we also provide a Nanopore R10.4.1 *E. coli* native DNA sample with a tandem repeat (ATGATGATG) in Supplementary Figure S8, which demonstrates the signals of tandem repeat are consistent.

In conclusion, nanoCEM provides multi-dimensional visualizations and statistical analysis for ONT DNA/RNA reads on different platforms. By utilizing rescaling and calculating various statistical features, nanoCEM provides feasible and accurate feature tables, allowing researchers to effectively observe differences between samples, and provide a framework for downstream training for DNA/RNA modification predictors.

## Supplementary Material

lqae052_Supplemental_File

## Data Availability

The *E. coli* 23S rRNA and HCC78 DNA raw sequencing files in our research are available at Figshare, including scWGA data (hg38 DNA reads on chr17): https://doi.org/10.6084/m9.figshare.25106996.v1, WGS data (hg38 DNA reads on chr17): https://doi.org/10.6084/m9.figshare.25106987.v1, IVT data (23S rRNA reads from *E. coli*): https://doi.org/10.6084/m9.figshare.25106936.v1, WT data (23S rRNA reads from *E. coli*): https://doi.org/10.6084/m9.figshare.25106945.v1

## Code availability

The pipeline for visualizing multiple features and conducting statistical analysis is available on GitHub at https://github.com/lrslab/nanoCEM and Zenodo at https://doi.org/10.5281/zenodo.11095704. Additionally, you can find the documentation for this pipeline at https://nanocem.readthedocs.io/en/latest/.
