# Supplementary material for: Nanopore Current Events Magnifier (nanoCEM): a novel tool for visualizing current events at modification sites of nanopore sequencing
Source: NAR Genom Bioinform. 2024 May 20;6(2):lqae052. doi: 10.1093/nargab/lqae052 (PMC11106030; doi:10.1093/nargab/lqae052)
Supplement: lqae052_Supplemental_File [file lqae052_supplemental_file.docx]

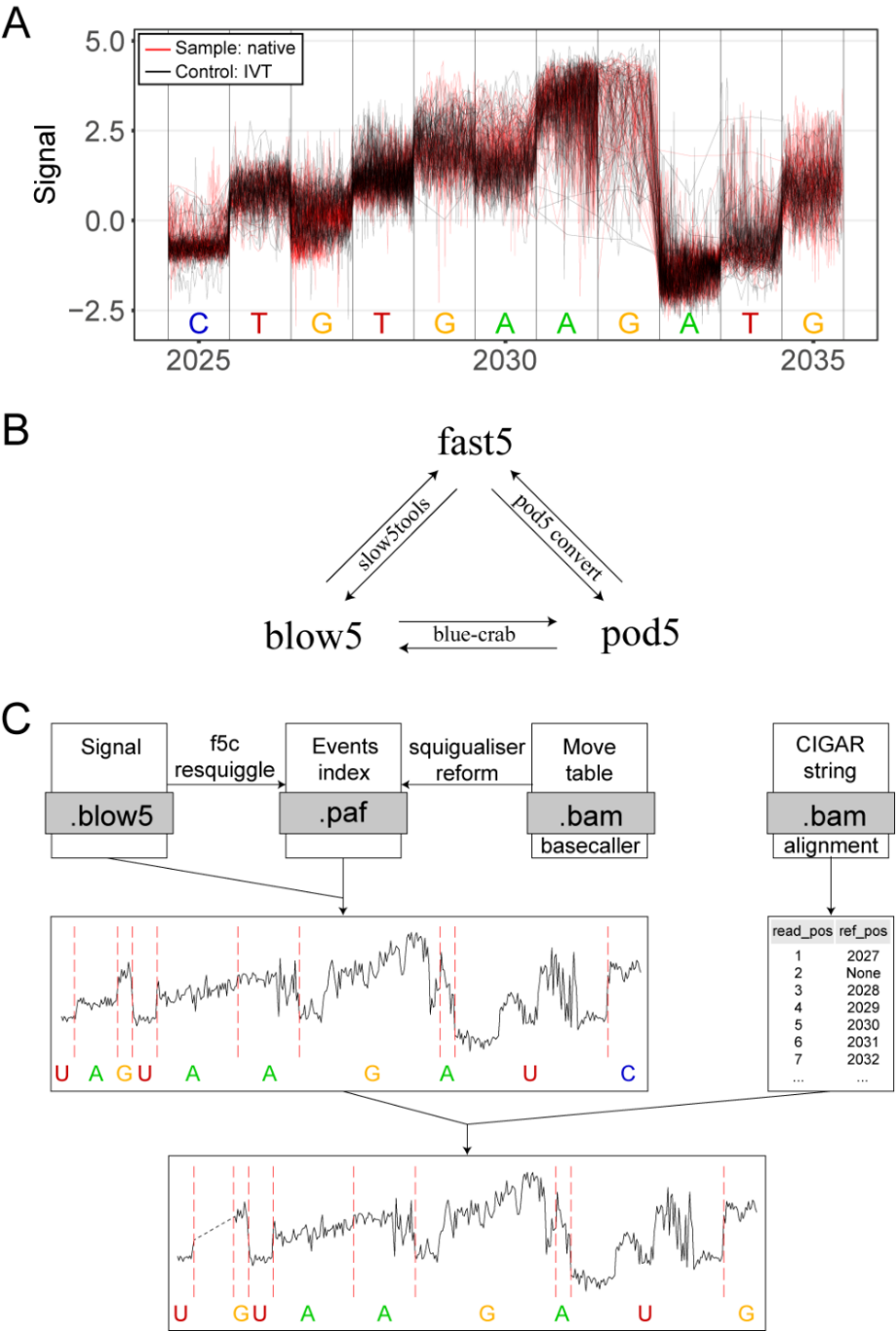


**Figure S1**. **A** The raw signal plot from Tombo plot, subsampling both the sample and control datasets to 50 reads each. **B** Major data formats and conversion methods for ONT sequencing. **C** The pipeline of tackling f5c resquiggle and move table using the reference genome.

**
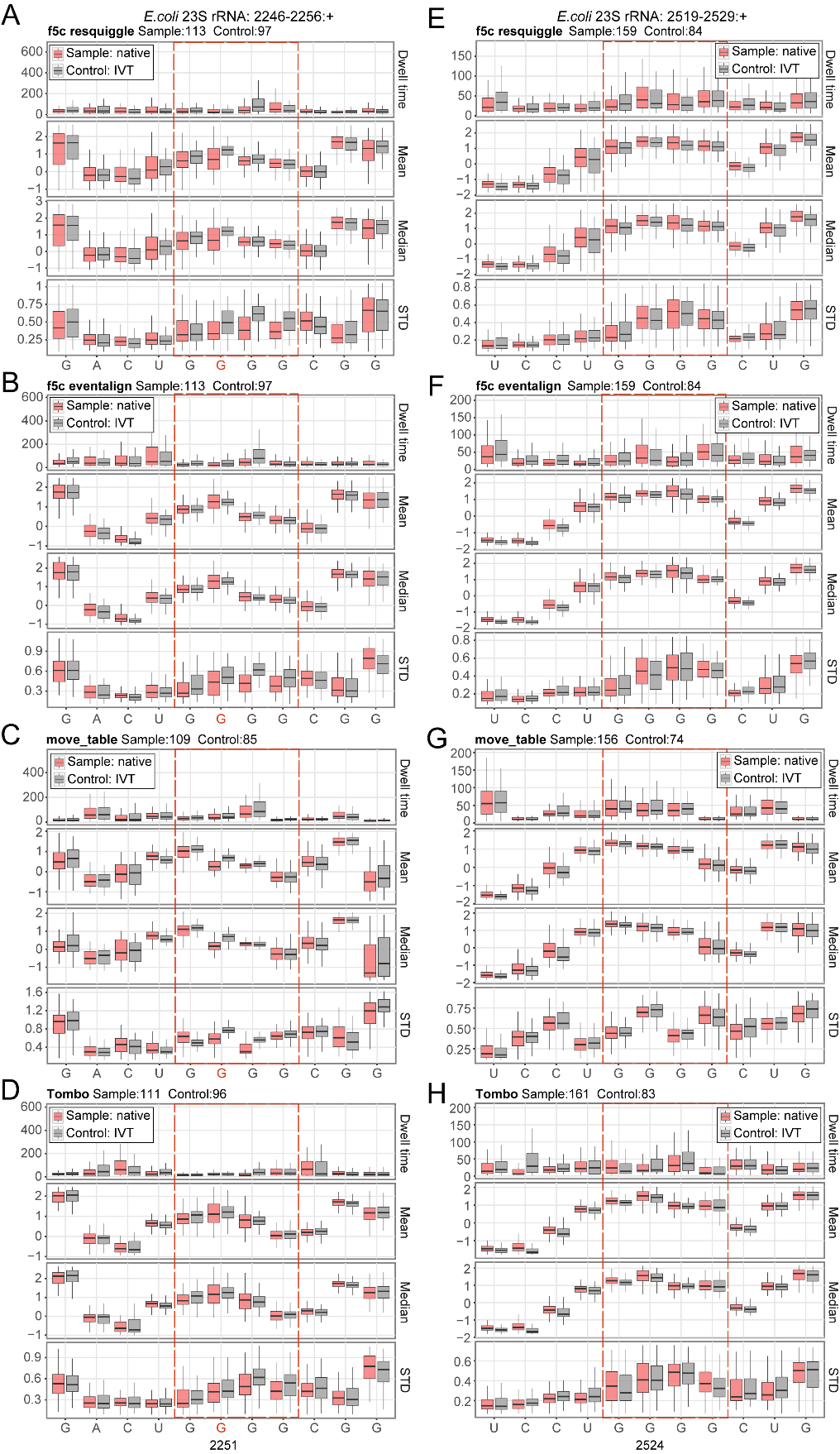
**

**Figure S2**. The nanoCEM features are shown on two polyN regions from *E. coli* 23S rRNA. The native and IVT RNA002 direct-RNA sequencing reads are indicated as red and grey. **A**, **B, C** and **D** are on a polyG region that includes G2251, a known 2'-O-methylation site in *E. coli* 23S rRNA. And **E, F, G, H** are on an unmodified ployG region (2523 to 2526). The two regions are highlighted by red boxes.


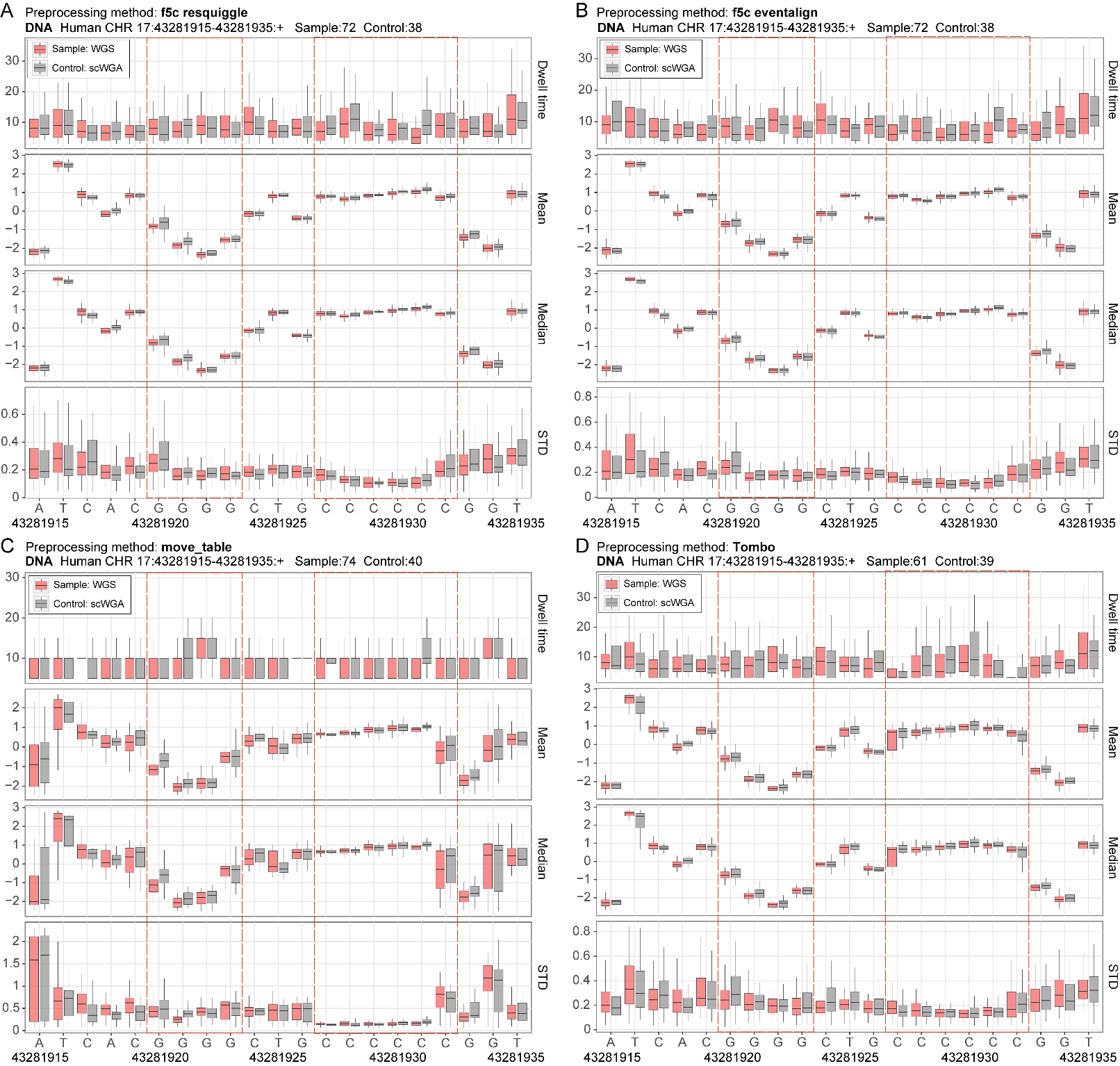
**Figure S3**. The nanoCEM features are shown on two polyN regions from human chromosome 17. The WGS and scWGA R9.4.1 DNA reads are indicated as red and grey. The polyG (43281920-43281923) and polyC (43281927-43281932) regions are highlighted by the red box.


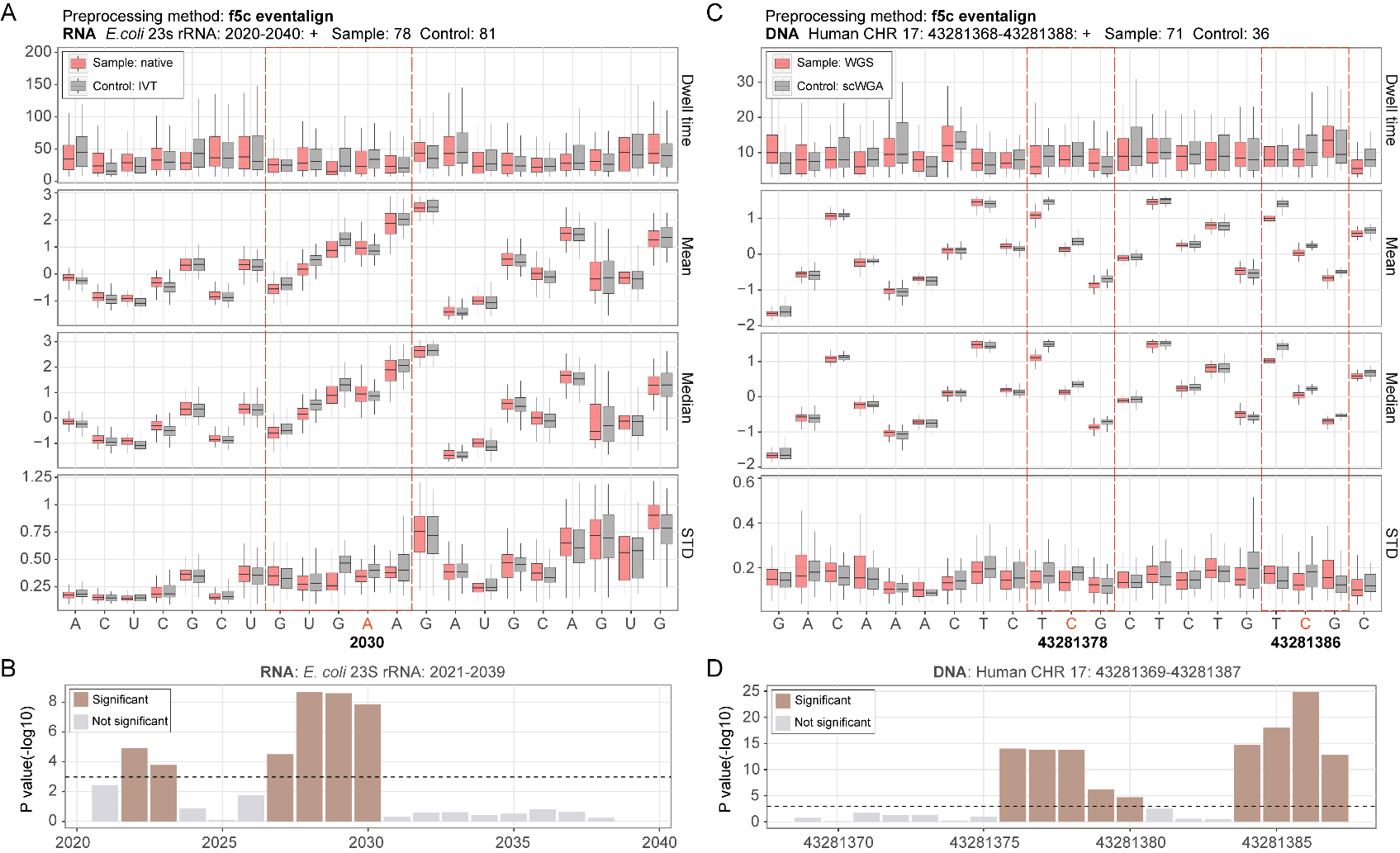
**Figure S4**. The implementation of nanoCEM with f5c eventalign preprocessing. **A** The current events feature visualization for the A2030 from native *E. coli* RNA (wild type sample) and IVT (in vitro transcribed RNA, serving as a negative control). The significant differences between negative and native samples are highlighted in the red box. And **B** represents the significance levels from MANOVA in the target region. The dashed line indicates a P value = 1e-3. **C** and **D** are for the two CpG sites from DNA sample comparing WGS and scWGA sequencing data.


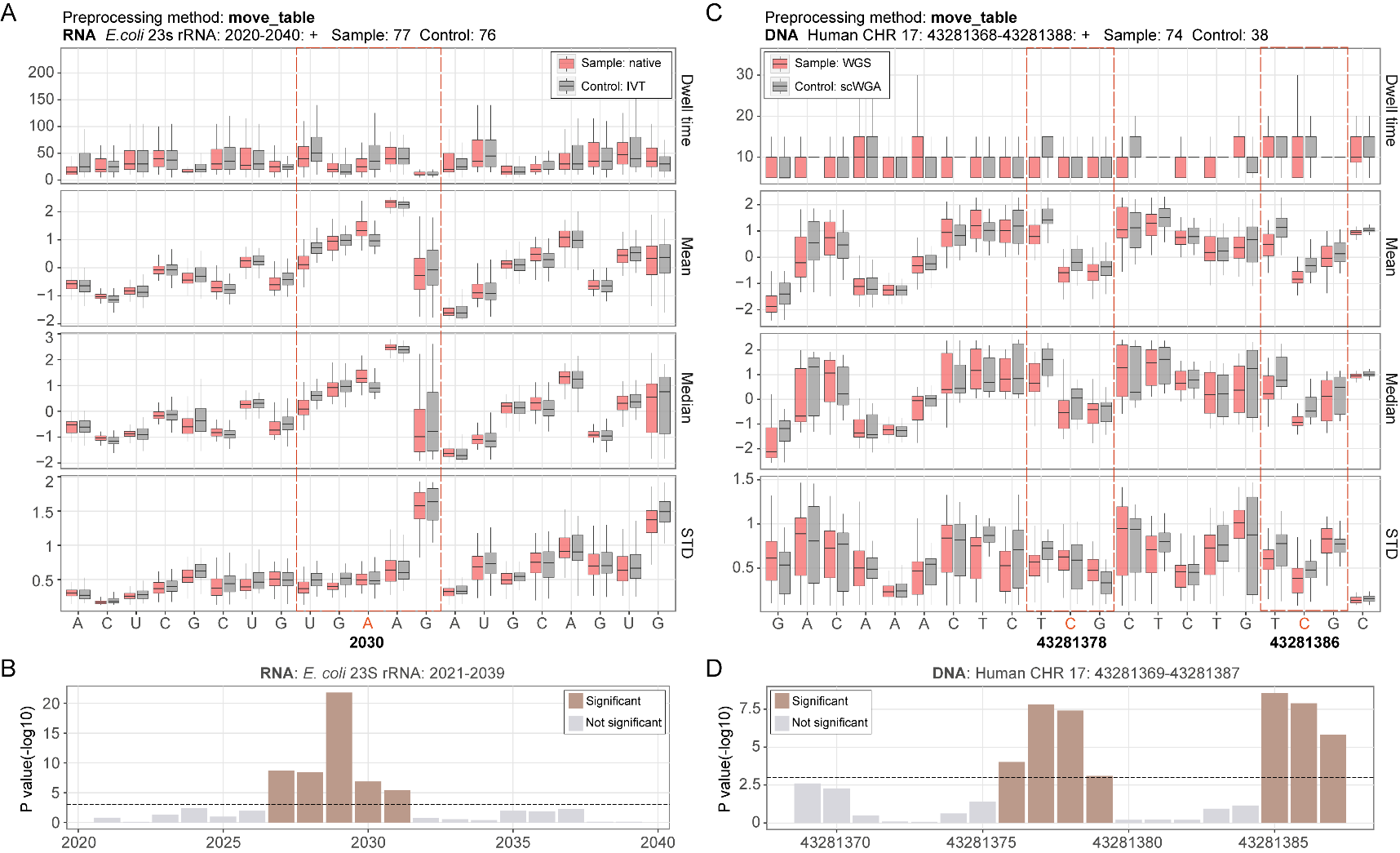
**Figure S5**. The implementation of nanoCEM with move table from ONT basecaller. **A** The current events feature visualization for the A2030 from native *E. coli* RNA (wild type sample) and IVT (in vitro transcribed RNA, serving as a negative control). The significant differences between negative and native samples are highlighted in the red box. And **B** represents the significance levels from MANOVA in the target region. The dashed line indicates a P value = 1e-3. **C** and **D** are for the two CpG sites from DNA sample comparing WGS and scWGA sequencing data.


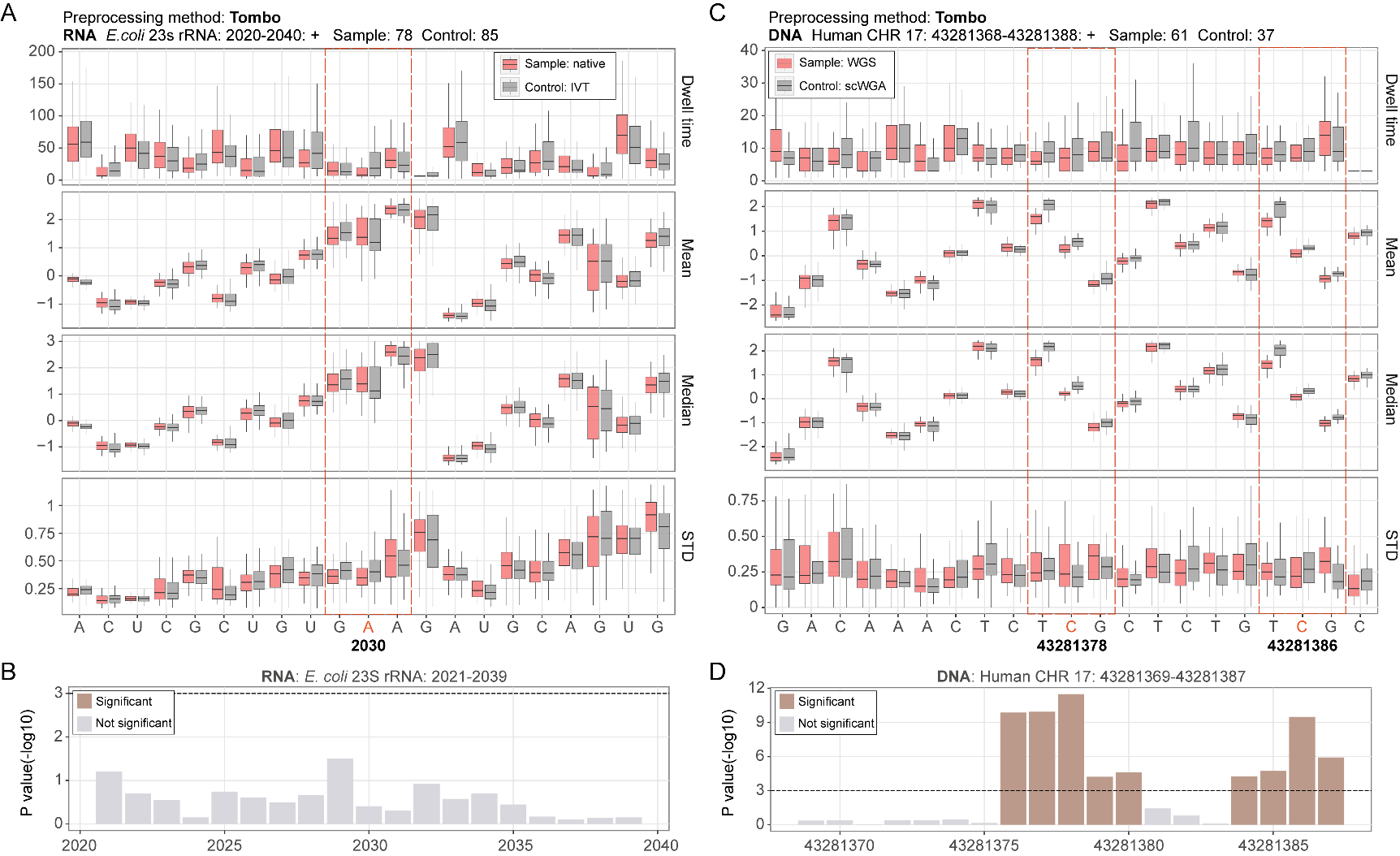
**Figure S6**. The implementation of nanoCEM with Tombo preprocessing. **A** The current events feature visualization for the A2030 from native *E. coli* RNA (wild type sample) and IVT (in vitro transcribed RNA, serving as a negative control). The significant differences between negative and native samples are highlighted in the red box. And **B** represents the significance levels from MANOVA in the target region. The dashed line indicates a P value = 1e-3. **C** and **D** are for the two CpG sites from DNA sample comparing WGS and scWGA sequencing data.


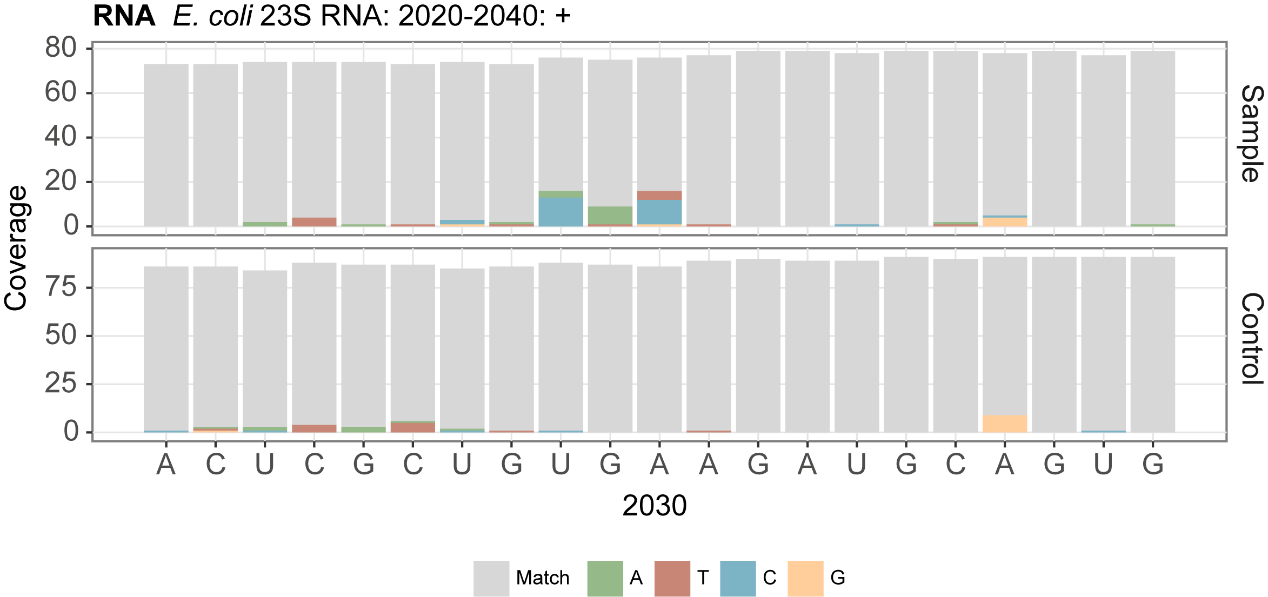


**Figure S7**. The alignment feature for ONT reads basecalled by dorado with model rna002_70bps_hac@v3.


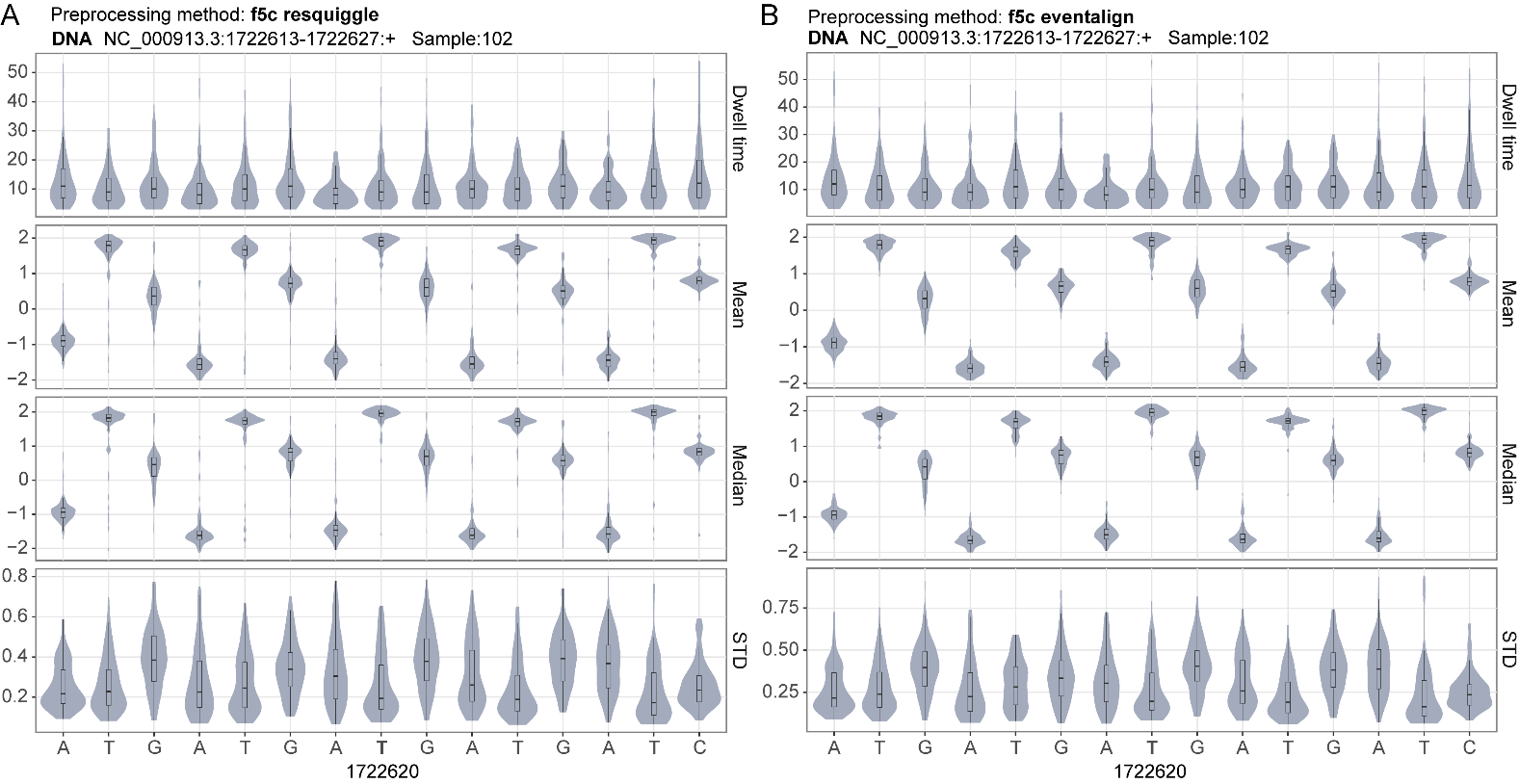
**Figure S8**. Current features for nanopore R10.4.1 *E. coli* native DNA sample inside a tandem repeat using nanoCEM single mode. **A** is derived from f5c resquiggle and **B** is f5c eventalign.
